# Retinal Fibre Layer Thickness Measurement in Normal Paediatric Population in Sweden Using Optical Coherence Tomography

**DOI:** 10.1155/2016/4160568

**Published:** 2016-11-17

**Authors:** Marcelo Ayala, Evangelia Ntoula

**Affiliations:** ^1^Eye Department, Skaraborg Hospital, Skövde, Sweden; ^2^Sahlgrenska Academy, Gothenburg University, Gothenburg, Sweden; ^3^Karolinska Institute, Solna, Sweden; ^4^Eye Department, Uppsala University Hospital, Uppsala, Sweden

## Abstract

*Purpose*. To evaluate the correlation between peripapillary retinal nerve fibre layer (RNFL) thickness and both age and refraction error in healthy children using optical coherence tomography (OCT).* Patients and Methods*. 80 healthy children with a mean age of 9.1 years (range 3.8 to 16.7 years) undergoing routine ocular examination at the orthoptic section of the Ophthalmology Department were recruited for this cross-sectional study. After applying cycloplegia, the peripapillary RNFL thickness was measured in both eyes using the Topcon 3D OCT 2000 device.* Results*. 138 eyes were included in the analysis. The average refractive error (SE) was +1.7 D (range −5.25 to +7.25 D). The mean total RNFL thickness was 105 *μ*m ± 10.3, the mean superior RNFL thickness was 112.7 *μ*m ± 16.5, and the mean inferior RNFL thickness was 132.6 *μ*m ± 18.3. We found no statistically significant effect of age on RNFL thickness (ANOVA, *f* = 0.33, *p* = 0.56). Refraction was proven to have a statistically significant effect (ANOVA, *f* = 67.1, *p* < 0.05) in RNFL measurements.* Conclusions*. Data obtained from this study may assist in establishing a normative database for a paediatric population. Refraction error should be taken into consideration due to its statistically significant correlation with RNFL thickness.

## 1. Introduction

Optical coherence tomography (OCT) is a noncontact, noninvasive optical imaging method that has the potential to create high resolution, live cross-sectional images of internal tissue structures similar to tissue sections under a microscope. Recent technical developments in the implementation of broadband light sources and the collection of frequency-encoded signals into OCT systems have allowed for improvements in scanning speed and axial resolution. OCT has evolved into an invaluable clinical tool in ophthalmology with a wide range of applications, though it has mainly been used for the detection and follow-up of retinal disease and glaucoma by means of measurement of macular thickness, optic head, and RNFL thickness [1–3].

Glaucoma and other optic neuropathies cause retinal ganglion cell damage, thus causing thinning of the RNFL. It has been reported that substantial retinal ganglion cell loss may have occurred at a specific location before corresponding visual field loss is detected [4]. As a result, evaluation of RNFL is fundamental in earlier detection of glaucoma and other optic neuropathies.

Various studies have proved the reproducibility and good diagnostic ability of OCT for both adults and children [5–8]. Nevertheless, all OCT manufacturers have an integrated normative database only for subjects of 18 years and older [9]. It is probable that these data cannot be extrapolated and applied to children in regard to the possible differences in the RNFL thickness between various age groups. Previous studies in adults have shown that the RNFL becomes thinner with advanced age [4, 10]. Several studies have also evaluated variation in RNFL thickness in the paediatric population, though these studies mainly used the previous TD-OCT devices [5, 11–14]. Similar reports using SD-OCT are less available in the literature.

The purpose of this study was to evaluate the correlation of RNFL thickness with age and refraction in healthy children and adolescents using the newer SD-OCT.

## 2. Materials and Methods

### 2.1. Subjects

This was a prospective cross-sectional study of healthy children aged 3.8 to 16.7 who visited the Paediatric Ophthalmology Section at the Ophthalmology Department of Skaraborg Hospital from September 2014 to September 2015.

The included group of children was referred to the Ophthalmology Department due to failed school or primary care visual screening, visual behaviour/abnormalities noticed by parents, or possible family history of refractive errors. Children at follow-up examination due to strabismus or amblyopia and in some cases healthy volunteers were also enrolled in this study. Ethics approval was obtained from the institutional review board (Ethical Approval: 717-13, Gothenburg, Sweden). Informed consent was obtained from parents or legal guardians.

### 2.2. Ocular Examination

All subjects underwent history-taking and routine ocular examination, which included visual examination using age-appropriate charts, motility examination, stereoacuity testing, slit lamp examination, cycloplegic refraction, and dilated fundoscopy. Pupils were dilated using cyclopentolate hydrochloride 0.85% + phenylephrine 1.5% eye drops (APL, Apotek, Sweden). Automatic refraction was performed 30–40 min. after the drops using the Auto Kerato-Refractometer KR-8800 (Topcon Corporation, Tokyo, Japan). Age, sex, and spherical equivalent refractive errors (SE) for each eye were recorded. The spherical equivalent was calculated by adding half the cylinder to the sphere of the average refractive values of each eye. SD-OCT analysis was then performed for each eye.

### 2.3. SD-OCT Imaging

Peripapillary SD-OCT RNFL thickness measurements were obtained using the Topcon 3D OCT 2000 device (Topcon Corporation, Tokyo, Japan). The protocol used was the 3D optic disc protocol (6 × 6 mm, 512 × 128) which generates images from 128 horizontal linear scans performed by 512 A-scans and measures the RNFL thickness in a 6 × 6 mm area around the optic disk. The scan speed is around 50,000 A-scans per second. In-depth resolution was about 5-6 *μ*m.

The RNFL values used in this study were generated automatically by the machine.

OCT images were obtained by the same operator through the dilated pupil. An internal fixation target was used while scanning and centring of the scans were confirmed by observing the optic disc on the video screen. The best centred image and the one of the best quality, determined by the signal strength index of the device, was chosen for analysis. For the purpose of this study, images with quality index below 60 were excluded. The signal strength index is given automatically by the machine and is based on image quality reflecting the reliability of the measurements performed.

Values for the average RNFL thickness, average superior, and inferior segment RNFL thickness were obtained in each series of scans.

### 2.4. Statistical Analysis

Associations between RNFL thickness, age, and refraction, spherical equivalent (SE), were studied by univariate linear regression analysis, with RNFL as the dependent variable and age or SE as independent variables. The Kolmogorov-Smirnov test for normality and Levene's test for homoscedasticity were performed. Analysis of variance (ANOVA) was used to test significance in the regression analysis (SPSS, Chicago, USA). Significance level is 0.05.

## 3. Results

### 3.1. Demographics

Eighty children were enrolled in this study, with an average age of 9.1 (range 3.8 to 16.7 years). Of these subjects, 45 were female and 35 were male. Overall, 160 eyes were examined and 138 eyes (86%) were included in the analysis. The results from 22 eyes were excluded from analysis due to either inadequately centred images or quality index lower than 60. Overall, 72 right eyes and 66 left eyes were included in the analysis. The mean refraction found was +1.7 D, with a range −5.25 to +7.5 D. With regard to ethnicity of the subjects included in the study, all children were born in Sweden. The majority (84%) had both parents originating from Sweden and 8% had one parent from Sweden and the other from abroad, while 8% had both parents from abroad.

### 3.2. RNFL Thickness Measurements

The mean total RNFL thickness was 105 *μ*m ± 10.3, the mean superior RNFL thickness was 112.7 *μ*m ± 16.5, and the mean inferior RNFL thickness was 132.6 *μ*m ± 18.3. The Kolmogorov-Smirnov test showed that the data were normally distributed (*p* = 0.07). Levene's test showed homoscedasticity (*p* = 0.36).

### 3.3. Effect of Age on RNFL Thickness

The RNFL thickness (total, superior, and inferior) showed no significant association with age in this population group. For the total RNFL thickness ANOVA, *f* = 0.33, *p* = 0.56. For the superior sector RNFL thickness, ANOVA, *f* value = 0.31, *p* = 0.57, and for the inferior sector RNFL thickness ANOVA, *f* value = 0.073, *p* = 0.08.

**Figure 1:**
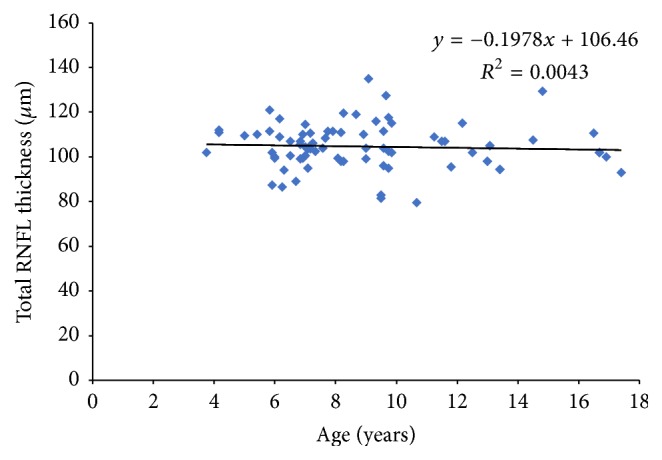
Scatter plots illustrating the relationship between total RNFL measurements and age.

**Figure 2:**
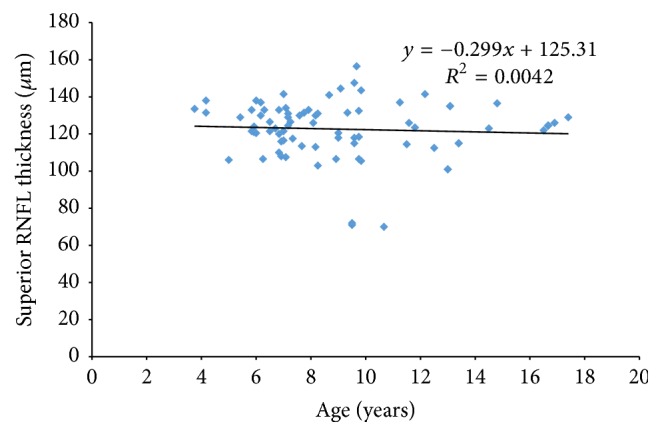
Scatter plots illustrating the relationship between superior RNFL measurements and age.

**Figure 3:**
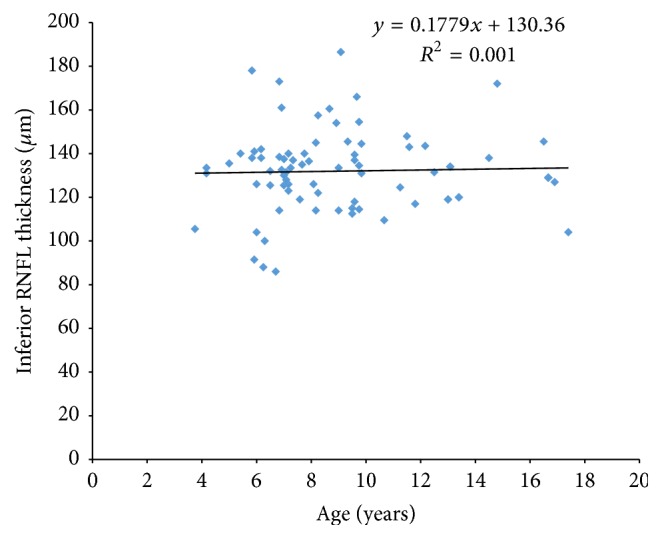
Scatter plots illustrating the relationship between inferior RNFL measurements and age.

Figures 1, 2, and 3 show the relationship between age and total, superior, and inferior RNFL thickness, respectively.

### 3.4. Effect of Refractive Error on RNFL Thickness

The spherical equivalent (SE) ranged from −5.25 to +7.5 D with a mean refractive error of 1.7 D. The SE was found to have a significant effect on RNFL thickness, with an increase by 2.1 *μ*m of the average total RNFL thickness for every diopter towards hyperopia.

Total RNFL thickness showed a significant association with SE, ANOVA, *f* = 67.1, *p* < 0.05. When compared among sectors, RNFL thickness was not positively correlated with SE in the superior sector, ANOVA, *f* = 0.66, *p* = 0.41. Meanwhile, the inferior sector showed a positive correlation with SE, ANOVA, *f* = 10.4, *p* < 0.05.

**Figure 4:**
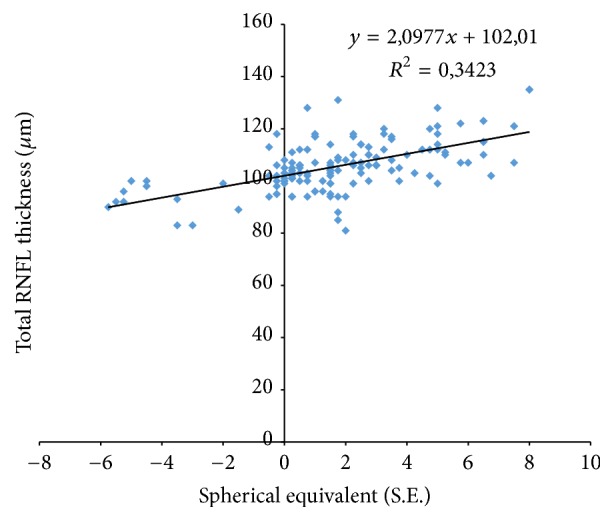
Scatter plots illustrating the relationship between total RNFL measurements and spherical equivalent (SE).

**Figure 5:**
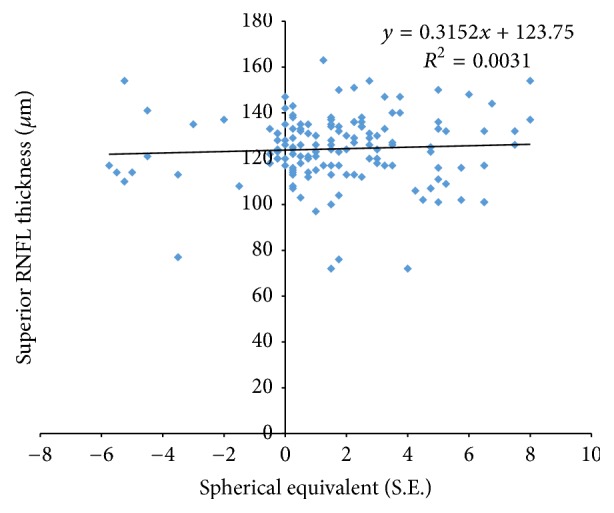
Scatter plots illustrating the relationship between superior RNFL measurements and spherical equivalent (SE).

**Figure 6:**
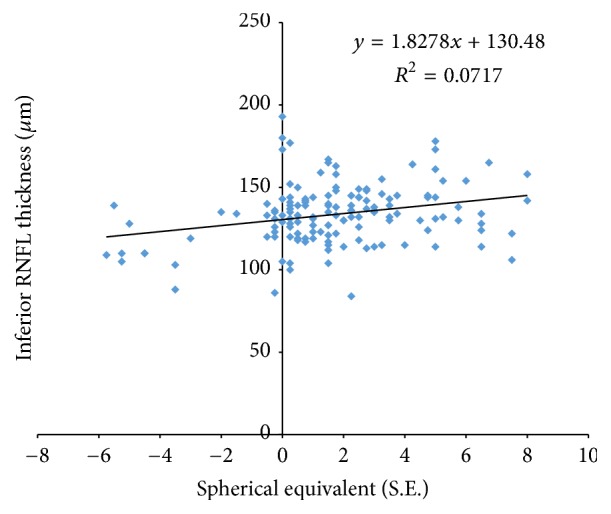
Scatter plots illustrating the relationship between inferior RNFL measurements and spherical equivalent (SE).

Figures 4, 5, and 6 show the relationship between SE and total, superior, and inferior RNFL thickness, respectively.

## 4. Discussion

SD-OCT is increasingly being used as a diagnostic and monitoring tool not only for adults but also for children, due to the short exposure duration and eye tracking systems in the latest devices allowing high quality images to be obtained.

In this study we investigated peripapillary RNFL thickness in normal children as measured using the latest version of SD-OCT, and we also determined the effect of age and refraction.

We found that the mean total RNFL thickness was 105 *μ*m ± 10.3, which is comparable to data obtained from previous studies [5, 12, 13, 15–17] and slightly higher compared to another group of studies using mainly the Cirrus OCT [6, 9, 18, 19]. It is possible that this discrepancy is due to segmentation differences in the definition of the outer border of RNFL and optical interaction with tissue due to different light sources and laser camera system sensor between Cirrus and Topcon OCT [20].

Table 1 compares our results with those from previous studies.

In our study we noticed that the mean inferior RNFL value was thicker compared to the mean superior RNFL value, which is in agreement with data obtained from previous studies [13, 15–18].

The effect of age on RNFL thickness has been widely discussed. In a study of 190 participants with an age range of 9–86 years, Alasil et al. [4] concluded that mean RNFL thickness values decrease significantly with age at a rate of 1.5–1.6 *μ*m per decade of life. The results from our study show no statistically significant correlation between the average total, superior, and inferior RNFL thickness and age, which is in agreement with similar studies in paediatric populations using both TD-OCT and SD-OCT [5, 9, 11, 12, 15–19].

Our results are in contrast to reported data from Salchow et al. [13], who found that age had a significant negative linear correlation with average RNFL thickness, but they concluded that age did not significantly affect RNFL thickness when adjusting for refractive error.

Age-related thinning of the RNFL is known; though it is unclear at what age the thinning starts. It is possible that the change in thickness in an individual case starts later in adult life, which may explain the lack of statistically significant correlation between RNFL thickness and age in our study. This was also in agreement with Parikh et al. [10], who, in a population study of 59 subjects (age range 5–75), noticed a decline of 0.16 *μ*m per year after the age of 50 that was not uniformly distributed in all quadrants.

The results of our study suggest that there is a statistically significant positive correlation between SE refractive error and average RNFL thickness, with an increase of 2.1 *μ*m in mean total RNFL thickness for every diopter towards hyperopia. Our findings are close to the results of other studies evaluating the effects of refractive errors on mean RNFL thickness [13, 15, 19]. Among these studies, Turk et al. [16] were the only one to report a lack of statistically significant correlation between SE and average RNFL thickness, which may be a result of the rather smaller range of SE (−4 to +4) included in the study.

The positive correlation between average RNFL thickness and SE also applied when the inferior sector was studied. Taş et al. [21], in a study evaluating the relationship between SE and RNFL thickness in high- and low-hyperopic children, concluded that children with high hyperopia had thicker average in total and inferior RNFL compared to those with low hyperopia. In addition, Lim and Chun [22] also reported lower average and inferior RNFL thickness in a group of high myopic children compared to low myopic children.

The thinner RNFL thickness in myopic eyes may be a result of mechanical eye globe elongation associated with myopia and therefore retinal stretching and thinning [15, 22]. Another possible explanation may be the effect of optical magnification, which can cause measurement artefacts [15]. In longer eyes, such as myopic eyes, the axial diameter of an OCT scan circle projected onto the retina is larger than the preset scan diameter, and the OCT scan circle is therefore further away from the optic disk margin, resulting in smaller measured values of RNFL thickness. However, the impact of optical magnification is arguable [15].

There are some limitations in our study. The age of the patients included was not homogeneously distributed in the sample. Patients were recruited from orthoptists at our department, where the age range is mainly 4–9 years. Our study is not population-based, as patients enrolled in it were patients who had been referred for eye examinations due to failed visual screening. As a result, there may be some selection bias and the results may not be directly applied to the general population. Nonetheless, the lack of other exclusion criteria makes the study as close as possible to a population-based one.

In addition, we did not measure the axial length of the eyes examined; therefore we could not verify the optical magnification effect as it has been reported in the studies mentioned above. Another limitation was that we did not perform repeated measurements to achieve the best possible quality of scans taken. Despite this, we consider that this was closer to the reality of an ophthalmological clinical praxis.

To conclude, OCT has been proven to be a fast and easy-to-use imaging technique, providing high quality images of the RNFL in the paediatric population. Further studies involving large groups of paediatric patients should be performed in order to establish a normative database, which could facilitate the use of OCT in the early diagnosis and follow-up of optic nerve diseases.

## Figures and Tables

**Table 1:** Reported RNFL thickness OCT measurements in normal children.

OCT	Author	Number subjects	Number eyes	Age (years)	RNFL average (*µ*m)	RNFL sup. (*µ*m)	RNFL inf. (*µ*m)
Spectralis	Turk, 2012	107	107	10.5 ± 2.9	106.4 ± 9.4		
Spectralis	Yanni, 2012	83	83	8.9	107.6 ± 1.2		
RTVue-100	Tsai, 2012	470	470	9.2	109.4 ± 10	133.9 ± 18.1	142.2 ± 19.5
Cirrus	Elía, 2012	344	344	9.2 ± 1.7	98.5 ± 10.8	123.6 ± 19.5	130.2 ± 18.1
Cirrus	Rao, 2013	74	148	10 ± 3.4	94 ± 10.9	DX: 124 ± 14 SIN: 125 ± 16	DX: 117 ± 15 SIN: 117 ± 14
Cirrus	Al-Haddad, 2013	108	108	10.7 ± 3.1	95.6 ± 8.7	120.6 ± 14	124.8 ± 18
Topcon 3D 2000	Our study, 2016	80	138	9.1 ± 3.3	105 ± 10.3	122.7 ± 16.5	132.6 ± 18.3

## References

[1] Yaqoob Z., Wu J., Yang C. (2005). Spectral domain optical coherence tomography: a better OCT imaging strategy. *BioTechniques*.

[2] Costa R. A., Skaf M., Melo L. A. S. (2006). Retinal assessment using optical coherence tomography. *Progress in Retinal and Eye Research*.

[3] Gabriele M. L., Wollstein G., Ishikawa H. (2011). Optical coherence tomography: history, current status, and laboratory work. *Investigative Ophthalmology & Visual Science*.

[4] Alasil T., Wang K., Keane P. A. (2013). Analysis of normal retinal nerve fiber layer thickness by age, sex, and race using spectral domain optical coherence tomography. *Journal of Glaucoma*.

[5] Pawar N., Maheshwari D., Ravindran M., Ramakrishnan R. (2014). Retinal nerve fiber layer thickness in normal Indian pediatric population measured with optical coherence tomography. *Indian Journal of Ophthalmology*.

[6] Altemir I., Pueyo V., Elía N., Polo V., Larrosa J. M., Oros D. (2013). Reproducibility of optical coherence tomography measurements in children. *American Journal of Ophthalmology*.

[7] Blumenthal E. Z., Williams J. M., Weinreb R. N., Girkin C. A., Berry C. C., Zangwill L. M. (2000). Reproducibility of nerve fiber layer thickness measurements by use of optical coherence tomography. *Ophthalmology*.

[8] Wang X. Y., Huynh S. C., Burlutsky G., Ip J., Stapleton F., Mitchell P. (2007). Reproducibility of and effect of magnification on optical coherence tomography measurements in children. *American Journal of Ophthalmology*.

[9] Al-Haddad C., Barikian A., Jaroudi M., Massoud V., Tamim H., Noureddin B. (2014). Spectral domain optical coherence tomography in children: normative data and biometric correlations. *BMC Ophthalmology*.

[10] Parikh R. S., Parikh S. R., Sekhar G. C., Prabakaran S., Babu J. G., Thomas R. (2007). Normal age-related decay of retinal nerve fiber layer thickness. *Ophthalmology*.

[11] Leung M. M. P., Huang R. Y. C., Lam A. K. C. (2010). Retinal nerve fiber layer thickness in normal Hong Kong Chinese children measured with optical coherence tomography. *Journal of Glaucoma*.

[12] El-Dairi M. A., Asrani S. G., Enyedi L. B., Freedman S. F. (2009). Optical coherence tomography in the eyes of normal children. *Archives of Ophthalmology*.

[13] Salchow D. J., Oleynikov Y. S., Chiang M. F. (2006). Retinal nerve fiber layer thickness in normal children measured with optical coherence tomography. *Ophthalmology*.

[14] Qian J., Wang W., Zhang X. (2011). Optical coherence tomography measurements of retinal nerve fiber layer thickness in Chinese children and teenagers. *Journal of Glaucoma*.

[15] Tsai D.-C., Huang N., Hwu J.-J., Jueng R.-N., Chou P. (2012). Estimating retinal nerve fiber layer thickness in normal schoolchildren with spectral-domain optical coherence tomography. *Japanese Journal of Ophthalmology*.

[16] Turk A., Ceylan O. M., Arici C. (2012). Evaluation of the nerve fiber layer and macula in the eyes of healthy children using spectral-domain optical coherence tomography. *American Journal of Ophthalmology*.

[17] Yanni S. E., Wang J., Cheng C. S. (2013). Normative reference ranges for the retinal nerve fiber layer, macula, and retinal layer thicknesses in children. *American Journal of Ophthalmology*.

[18] Elía N., Pueyo V., Altemir I., Oros D., Pablo L. E. (2012). Normal reference ranges of optical coherence tomography parameters in childhood. *British Journal of Ophthalmology*.

[19] Rao A., Sahoo B., Kumar M., Varshney G., Kumar R. (2013). Retinal nerve fiber layer thickness in children <18 years by spectral-domain optical coherence tomography. *Seminars in Ophthalmology*.

[20] Pierro L., Gagliardi M., Iuliano L., Ambrosi A., Bandello F. (2012). Retinal nerve fiber layer thickness reproducibility using seven different OCT instruments. *Investigative Ophthalmology and Visual Science*.

[21] Taş M., Oner V., Türkcü F. M. (2012). Peripapillary retinal nerve fiber layer thickness in hyperopic children. *Optometry and Vision Science Journal*.

[22] Lim H.-T., Chun B. Y. (2013). Comparison of OCT measurements between high myopic and low myopic children. *Optometry and Vision Science*.

